# Association of Cumulative Paternal Early Life Stress With White Matter Maturation in Newborns

**DOI:** 10.1001/jamanetworkopen.2020.24832

**Published:** 2020-11-24

**Authors:** Hasse Karlsson, Harri Merisaari, Linnea Karlsson, Noora M. Scheinin, Riitta Parkkola, Jani Saunavaara, Tuire Lähdesmäki, Satu J. Lehtola, Maria Keskinen, Juho Pelto, John D. Lewis, Jetro J. Tuulari

**Affiliations:** 1 FinnBrain Birth Cohort Study, Turku Brain and Mind Center, Institute of Clinical Medicine, University of Turku, Turku, Finland; 2 Department of Psychiatry, University of Turku and Turku University Hospital, Turku, Finland; 3 Department of Medical Physics, Turku University Hospital, Turku, Finland; 4 Department of Child Psychiatry, University of Turku and Turku University Hospital, Turku, Finland; 5 Department of Radiology, University of Turku and Turku University Hospital, Turku, Finland; 6 Department of Pediatric Neurology, Turku University Hospital and University of Turku, Turku, Finland; 7 McGill Centre for Integrative Neuroscience, Montreal Neurological Institute, McGill University, Montreal, Quebec, Canada; 8 Turku Collegium for Science and Medicine, University of Turku, Turku, Finland

## Abstract

**Importance:**

Early life stress (ELS) has been shown to affect brain development and health outcomes. Recent animal studies have linked paternal early stress exposures with next-generation outcomes. Epigenetic inheritance through the male germline has been suggested to be one of the mechanisms.

**Objectives:**

To test whether paternal ELS, as measured using the Trauma and Distress Scale, is associated with neonate brain development.

**Design, Setting, and Participants:**

This cohort study included data from participants from the prospective 2-generation FinnBrain Birth Cohort, which was collected from 2011 to 2015. Pregnant women and the fathers were consecutively recruited at gestational week 12 from maternity clinics in Finland. Magnetic resonance imaging data were analyzed in 2019. Participants in this study were 72 families (infant, father, mother).

**Exposure:**

Paternal exposure to ELS.

**Main Outcomes and Measures:**

Fractional anisotropy (FA) values in the major white-matter tracts of the newborn brain.

**Results:**

A total of 72 trios (infant, mother, and father) were analyzed. At the time of delivery, the mean (SD) age was 31.0 (4.4) years for fathers and 30.3 (4.5) years for mothers. Forty-one infants (57%) were boys; mean (SD) child age at inclusion was 26.9 (7.2) days from birth and 205 (8) days from estimated conception. Increasing levels of paternal ELS were associated with higher FA values in the newborn brain in the body of the corpus callosum, right superior corona radiata, and retrolenticular parts of the internal capsule. This association persisted after controlling for maternal ELS, maternal socioeconomic status (SES), maternal body mass index, maternal depressive symptoms during pregnancy, child sex, and child age from birth and gestation corrected age when imaged. In additional region-of-interest analyses, the association between FA values and paternal Trauma and Distress Scale sum scores remained statistically significant in the earliest maturing regions of the brain, eg, the genu of the corpus callosum (in the regression models, β = 0.00096; 95% CI, 0.00034-0.00158; *P* = .003) and the splenium (β = 0.00090; 95% CI, 0.00000-0.00180; *P* = .049).

**Conclusions and Relevance:**

This cohort study found a statistically significant association between paternal ELS and offspring brain development. This finding may have far-reaching implications in pediatrics, as it suggests the possibility of a novel route of intergenerational inheritance of ELS on next-generation brain development.

## Key Points

**Question:**

Is there an association between paternal early life stress exposure and newborn offspring brain development?

**Findings:**

In the FinnBrain Birth Cohort study, among 72 trios of infants and their parents, a statistically significant association was found between paternal cumulative early life stress and child brain development, which persisted after controlling for several maternal variables.

**Meaning:**

These data suggest an intergenerational mode of inheritance of offspring brain development; this finding may have implications for pediatric neuropsychiatric disorders.

## Teaser

This cohort study examines infants and parents in the FinnBrain Birth Cohort to assess the association between paternal cumulative early life stress and offspring brain development.

## Introduction

There is considerable evidence that exposure to early life stress (ELS) can have health consequences that may persist over the lifetime. Early life stress sets in motion irreversible developmental trajectories by influencing the psychobiological programming of the developing brain^1,2^ with negative health consequences persisting to adulthood.^3,4^ In addition to the stress the child experiences, exposure to maternal stress during pregnancy reportedly influences offspring development and health.^5,6^

In animal studies, a transgenerational epigenetic inheritance of paternal stress through the male germline via changes in sperm microRNAs also has been reported.^7,8^ In rodents, paternal stress seems to be reflected in changes in their offspring in both brain structure and function as well as behavior.^9,10^ If this association is also present in humans, it would have far-reaching implications, as this mechanism would allow the transfer of some of the effects of ELS to the offspring without the offspring being directly exposed to ELS.^11^

To the best of our knowledge, no human studies to date have tested whether paternal ELS experiences are associated with their infants’ brain development. In humans, causal mechanisms between parents’ own ELS and later offspring outcomes are difficult to delineate, as cultural transmission through parental nurturing and social patterning play a major role in child development.^12^ Our study was designed to minimize the role of the infant’s own exposure to postnatal ELS or other effects of postnatal life by acquiring neonate brain imaging data.

Within the current study, we acquired magnetic resonance imaging data in newborns and assessed white-matter development via fractional anisotropy (FA) in diffusion tensor imaging (DTI) data, the most widely accepted measure of white-matter integrity. Questionnaire and register data allowed controlling for many of the maternal and pregnancy-related variables. Our hypothesis was that paternal ELS is associated with their offspring’s early brain white matter development.

## Methods

The FinnBrain Birth Cohort Study (http://www.finnbrain.fi) was established in 2011 to prospectively investigate the effects of ELS, including prenatal stress exposure, on child brain development and health.^13^ The aim was to identify biomarkers associated with ELS exposures as well as eventual trajectories for common psychiatric and somatic illnesses. Families (pregnant women and the fathers) were consecutively recruited at gestational week (gwk) 12 from maternity clinics in southwest Finland from 2011 to 2015.

For brain imaging, 180 infants at aged 2 to 5 weeks were recruited by contacting the families via telephone (by study nurse or investigators). The age of the children was corrected for gestational age. Exclusion criteria for infants were occurrence of any perinatal complications with potential neurological consequences (eg, hypoxia), less than 5 points in the 5-minute Apgar score, previously diagnosed central nervous system anomaly or prior clinical magnetic resonance scan at peripartum owing to clinical indications, gestational age of less than 32 weeks, or birth weight less than 1500 g. These criteria were confirmed through a structured phone interview. Families were provided oral and written information about the study, and the parents provided written consent to participate on behalf of their child. The study was conducted in accordance with the Declaration of Helsinki^14^ and was approved by the Ethics Committee of the Hospital District of Southwest Finland (15.3.2011 §95, ETMK: 31/180/2011). We followed the Strengthening the Reporting of Observational Studies in Epidemiology (STROBE) reporting guideline.

As a part of the main FinnBrain Cohort, information about the parents’ childhood and adolescence ELS experience was collected using the Trauma and Distress Scale (TADS)^15^ at gwk 12. The TADS comprises 5 core domains: emotional neglect, emotional abuse, physical neglect, physical abuse, and sexual abuse. In this study, we calculated the cumulative exposure to ELS events of the infants’ fathers and mothers by the age of 18 years.

Maternal depressive symptoms during pregnancy were measured using the Edinburgh Postnatal Depressive Scale (EPDS)^16^ at gwk 24. Maternal measures of body mass index (BMI) and level of education (low: up to 12 years of education; middle: 12-15 years; and high: >15 years) were available from questionnaires and the Finnish Medical Birth Registry held by the National Institute for Health and Welfare.

**Figure 1.**
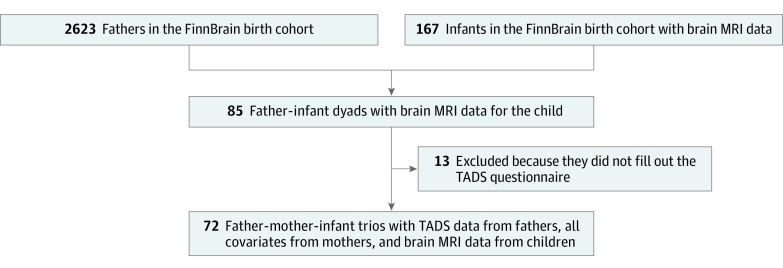
Flowchart of the Study MRI indicates magnetic resonance imaging; TADS, Trauma and Distress Scale.

We were able to acquire DTI data from 167 of 180 infant participants. Paternal and maternal data, including their TADS scores and relevant background data, were available for 72 trios (fathers, mothers, and infants). The final sample is described in Table 1. There was missing information (with 1 to 6 values missing; see Table 1 footnotes) in the following maternal variables: level of education, TADS score, EPDS, prepregnancy BMI, use of SSRIs and SNRIs, alcohol use, and mode of delivery. Missing information was imputed using mean (continuous variables) or mode (categorical variables) values of the variables. The flowchart of how the final sample was reached is shown in Figure 1.

**Table 1.** The Characteristics of the Study Population (N = 72)

Characteristic	No. (%)
Paternal age at the time of delivery, mean (SD), y	31.0 (4.4)
Paternal TADS, total sum score, mean (SD)	9.39 (8.86)
Paternal level of education	
Low	29 (40)
Middle	24 (33)
High	19 (26)
Maternal age at the time of delivery, mean (SD), y	30.3 (4.5)
Maternal TADS, total sum score, mean (SD)^a^	8.00 (7.35)
Maternal level of education^b^	
Low	17 (24)
Middle	24 (34)
High	29 (41)
Maternal EPDS, total sum score at gestational wk 24, mean (SD)^c^	5.38 (4.65)
Maternal BMI, mean (SD)^c^	25.0 (3.7)
SSRI/SNRI medication^d^	
No	57 (86)
Yes	9 (14)
Smoking	
No	69 (96)
Yes	3 (4)
Alcohol use^e^	
No	61 (90)
Yes	7 (10)
Gestational wk at time of birth	
Mean (SD) wk	39.8 (1.2)
Full term	71 (99)
Preterm (<37 wk)	1 (1)
Child sex	
Boy	41 (57)
Girl	31 (43)
Child age, mean (SD), d	
From estimated conception	305 (8)
From birth	26.9 (7.2)
FA value	
In body	0.317 (0.026)
In genu	0.357 (0.024)
In splenium	0.427 (0.034)
Apgar score	
1 min	8.42 (1.52)
5 min	8.94 (.99)
Birthweight, mean (SD), g	3542 (467)
Head circumference, mean (SD), cm	35.0 (1.3)
Mode of delivery^c^	
Vaginal delivery	49 (69)
Assisted vaginal delivery	10 (14)
Cesarean delivery	
Elective	3 (4)
Urgent	7 (10)
Emergency	2 (3)
Incidental findings	6 (8)
Preeclampsia	0
Gestational diabetes	10 (14)
Child antibiotic treatment	2 (3)

Abbreviations: BMI, body mass index (calculated as weight in kilograms divided by height in meters squared); EPDS, Edinburgh Postnatal Depressive Scale; FA, fractional anisotrophy; SNRI, selective serotonin-norepinephrine reuptake inhibitors; SSRI, selective serotonin reuptake inhibitor; TADS, Trauma and Distress Scale.

^a^ Missing 3 values.

^b^ Missing 2 values.

^c^ Missing 1 value.

^d^ Missing 6 values.

^e^ Missing 4 values.

Seven mothers reported drinking 1 to 2 drinks more seldom than monthly. Three mothers reported smoking 5 to 10 cigarettes per day; other smokers reported quitting when they became pregnant. There was no reported illicit drug use in the current sample. Seven mothers reported using selective serotonin reuptake inhibitors or selective serotonin-norepinephrine reuptake inhibitors during pregnancy. After confirming that these factors did not affect the results by replicating the analyses without them, these individuals were not excluded from the analysis.

The scanning site was the Medical Imaging Centre of the Hospital District of Southwest Finland). Before scanning, the infants were fed with breastmilk or formula until they slept and were subsequently gently swaddled into a vacuum mattress. No anesthetics were used.

All infants were provided with double hearing protection (wax plugs and custom-sized ear muffs). Standard ear muffs were given to parents, as they usually stayed in the scanning room throughout the scanning session. The personnel observed the scanning procedure through the window of the control room. A microphone and loudspeaker in the scanner room facilitated audio monitoring of the infant and contact with the parents. If the infant woke up during the scan, the session was discontinued.

Each set of structural infant images was checked by an experienced neuroradiologist (R.P.) to detect any possible incidental findings in the scans. In accordance with our study protocol, radiology reports were delivered to the researchers who then communicated them to the family within 1 to 4 weeks of the scans. In the case of an incidental finding, the parents were referred to a child neurologist (T.L.) for a neurological checkup (n = 6). These incidental findings were minor and not unusual to see in newborns,^17^ and they did not affect the imaging metrics of interest. Thus, we did not exclude participants based on these findings. None of the infants had any clinically identified, significant neurological symptoms or deficits in neurological development at the time of the neurological examination.

Magnetic resonance imaging scans were conducted on a Siemens Magnetom Verio 3T scanner (Siemens Medical Solutions) using a 12-element Head Matrix coil. We acquired multicontrast images, but only diffusion-weighted imaging data were used in this study. The total duration of the complete scanning protocol did not exceed 60 minutes, and the imaging was performed during natural sleep of the infant.

The diffusion-weighted imaging sequences were designed to maximize the success of data acquisition even with substantial motion. A 96-direction protocol was divided into 3 sequences (with 31, 32, and 33 diffusion encoding directions). This protocol made it possible to repeat just 1 of the sequences in case of significant subject motion during the scan. The 3 sequences consisted of spin echo-echo planar imaging sequences at 2-mm isotropic resolution (FOV, 208 mm; 64 slices; TR, 8500 ms; TE, 90 ms). Each of the 3 sequences contained 3 b0 images.

First, the b0 volumes were visually checked by 3 raters (J.J.T. and PhD students under his supervision) to assure that a b0 not corrupted by motion artifacts existed. The best of the b0 volumes was moved to the front of the 4D series, and the rest were removed. A brain mask was then created from the selected b0 volume with FSL’s (FMRIB Software Library, version 5.0.9)^18^ Brain Extraction Tool.^19^ The brain mask was visually inspected by 2 raters (J.J.T. and H.M.) to ensure that this step was successful.

Second, the quality of diffusion data sets were quantitatively evaluated using DTIPrep, which is an open-source software platform for quality control of dMRI data.^20^ DTIPrep automatically identifies multiple types of artifacts in 4D diffusion data. DTIPrep was run with default settings and was used only to detect poor-quality data before subsequent modeling. Directions and volumes that DTIPrep suggested as being of bad quality were discarded in later steps. Guided by the amount of high-quality diffusion encoding directions in the data, we used only the best of the 3 acquired sequences in subsequent analyses. Only data sets with a successful acquisition in 1 of the 3 parts and more than 26 out of 31 to 33 acquired diffusion encoding directions remaining were processed further and included in the analysis, a choice supported by prior validation studies.^21,22^ With this choice, we lost no trios from later analysis.

Third, the data were corrected for motion and eddy currents using FSL’s eddy tool.^23^ The transform parameters were used to align the 4D diffusion volumes, and the movement components were applied to the directional vectors. The resulting data were then examined to ensure that the remaining directions were evenly distributed.^24^ Finally, the 4D diffusion data set was processed with FSL’s dtifit, using the brain mask to limit the modeling to brain tissue only.

### Statistical Analysis

We chose to study FA, which is one of the most widely used DTI metrics and reflects the integrity of white-matter structure. Fractional anisotropy is a composite measure of the directionality of diffusion and eigenvalues, ranging between 0 and 1, with 0 being complete isotropic diffusion and 1 being highly directional diffusion.^25^

For further processing, we used the Tract-Based Spatial Statistics (TBSS) pipeline of FSL.^26^ The TBSS limits the analysis only to the skeletons of white-matter tracts, estimated from individual images, that are projected to a common skeleton space prior to statistical testing. Unlike conventional voxel-based analyses, TBSS does not require perfect brain-to-brain alignment or spatial smoothing, and the estimation of tract center decreases partial volume effects. As preprocessing options, we used the “tbss_2_reg -n” option that identifies the most representative image, which is then used as the target image for the linear and nonlinear transformations for all the other data to create a study-specific template. For convenience of displaying the results, the template was reoriented to a straight position along the anterior commissure–posterior commissure line. We then ran a modified version of the “tbss_3_postreg -S” step to incorporate registrations to the study-specific template and upsample the data to 1 mm^3^ resolution as per TBSS defaults. We used an FA threshold of 0.15 in the “tbss_4_prestats” module to create an FA skeleton.

We then performed voxel-based statistical analyses using FSL’s randomize tool^27^ and 2D optimized threshold-free cluster enhancement with 5000 permutations.^28^ First, we entered the paternal TADS sum scores as the explanatory variable in a general linear model and controlled for infant sex, infant age from conception at scan, infant age from birth at scan, maternal prenatal depressive symptoms (EPDS scores at gwk 24), maternal TADS sum score, maternal educational level, and prepregnancy maternal BMI. The models were corrected for multiple comparisons with threshold-free cluster enhancement, at *P* < .05 across the whole brain FA skeleton (which included 24 593 voxels). The regional FA values of the corpus callosum (CC) as defined by the infant JHU atlas were used to further visualize the finding of the whole-brain analyses.

Although we regarded the TBSS whole-brain results as our primary result, we also performed region-of-interest (ROI) analyses to provide complementary statistical testing that includes effect size estimates and other results that are not readily available in FSL’s output. To this end, we performed an automated, skeleton-based, ROI delineation (the values were extracted from the TBSS skeleton). We warped the JHU white-matter atlas^29^ to the infant mean FA space and used it to mask the genu, body, and splenium of the CC within the skeleton, and the mean FA values within all CC components were calculated.

We first calculated the zero order correlations (Pearson *r*) among the different regional FA values of the CC (genu, body, and splenium) and between the regional FA values and child age from conception and birth. Furthermore, possible sex differences in regional FA values were checked using *t* tests. After the preliminary examinations, we fitted a simple linear regression model (model 1) for each regional FA value (with FA value as the response) to test whether the regional FA values were associated with the paternal TADS scores. Next, we added child sex and its interaction with the TADS score to the models (model 2) to test whether the associations between the FA values and volumes and TADS scores were dependent on child sex. Last, we tested whether the associations persisted even after controlling for child sex, child age from conception, child age from birth, maternal prenatal stress measured using the EPDS, maternal TADS sum score, maternal education, maternal BMI, and maternal age (model 3). Some more complex polynomial associations between the FA values and paternal TADS scores were also tried, but the models with a simple linear association gave the lowest Akaike information criterion and were therefore chosen. One-sided *P* < .05 indicated statistical significance. All ROI analyses were conducted using R, version 3.6.3 (R Foundation) in 2019.

## Results

A total of 72 trios (infant, mother, and father) were analyzed. At the time of delivery, the mean (SD) age was 31.0 (4.4) years for fathers and 30.3 (4.5) years for mothers. Forty-one infants (57%) were boys; mean (SD) child age at inclusion was 26.9 (7.2) days from birth and 205 (8) days from estimated conception.

**Figure 2.**
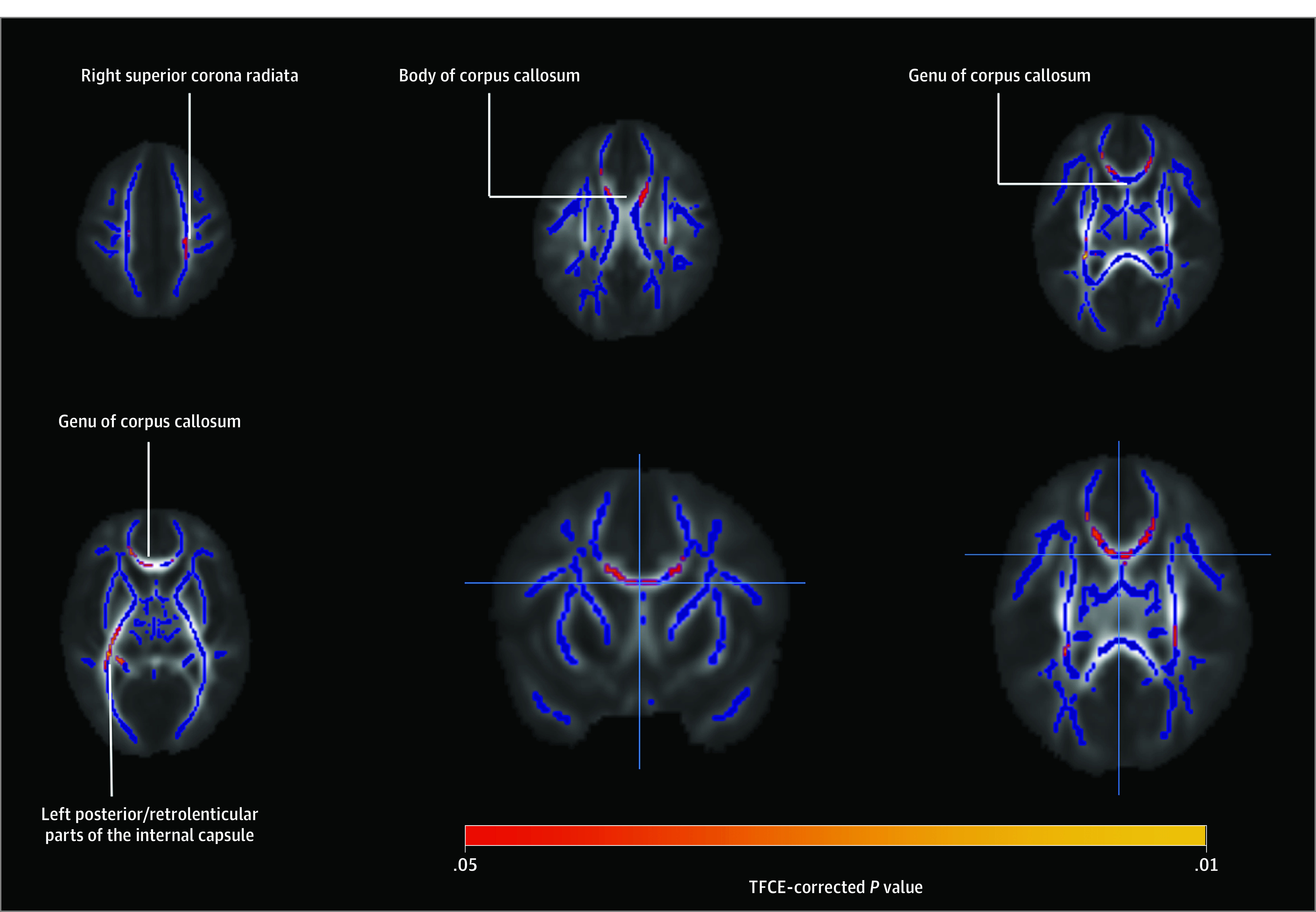
Affected White-Matter Tracts in the Brain Positive associations between infant fractional anisotropy values and paternal Trauma and Distress Scale (TADS) score in the voxel-based general linear model while controlling for infant sex and age from conception, maternal BMI, maternal depressive symptoms (Edinburgh Postnatal Depressive Scale score) at gestational week 24, and maternal TADS score. The statistical map has been overlaid on the FA skeleton, which includes only voxels with FA greater than 0.15. The study-specific infant template appears in the background. Only voxels reaching statistical significance (*P* < .05) are displayed, with threshold-free cluster enhancement corrected for multiple comparisons. The color bar shows the plotted *P* values. All figures are presented in neurological convention (left is left and right is right). Anatomical labels have been provided as per the JHU atlas (see the Methods for region of interest delineation).

**Figure 3.**
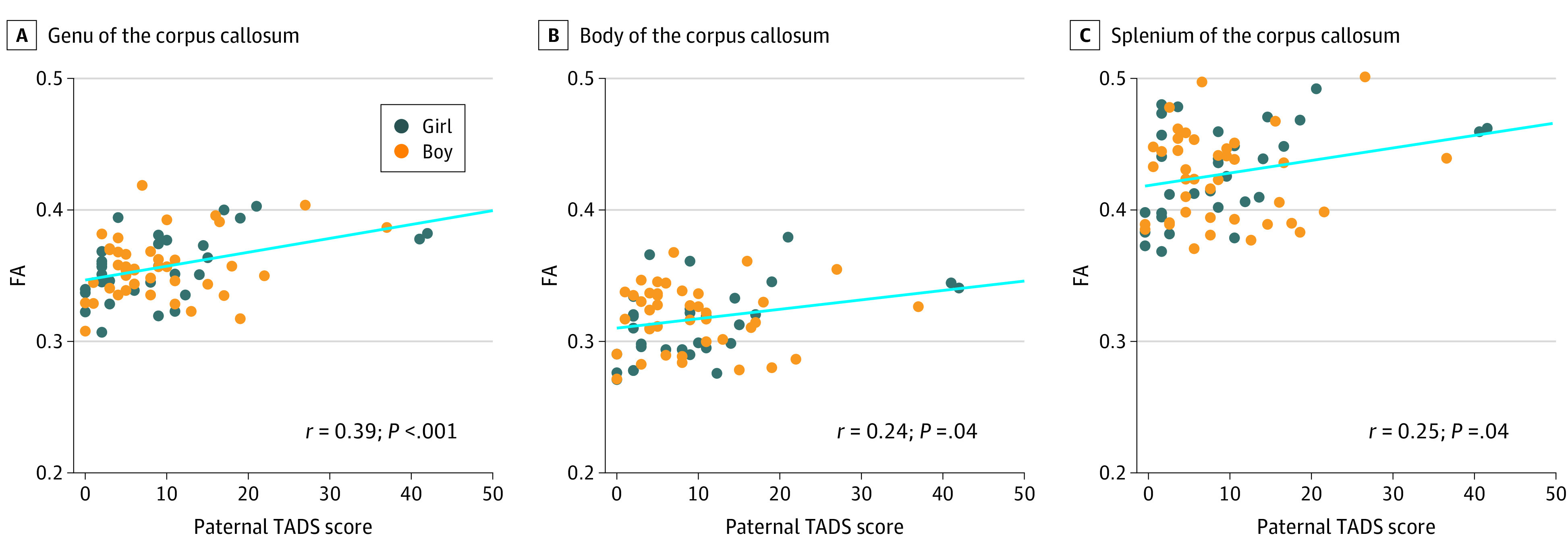
Trauma and Distress Scale (TADS) Scores and Corpus Callosum White-Matter Tracts Scatter plots and Pearson correlation coefficients of the association between paternal TADS sum scores and newborn fractional anisotropy (FA) values for 3 main corpus callosum subregions.

In the whole-brain regression model, we found a positive association between paternal early life stress (cumulative TADS scores) and infant FA values in the genu and body of the CC, the right superior corona radiata, and the left posterior and retrolenticular parts of the internal capsule while controlling fro maternal BMI, maternal EPDS score, and maternal TADS score (Figure 2). We then used the ROI approach to visualize the associations without controlling for any variables (Figure 3). The association between paternal TADS scores and infant FA values remained significant even after controlling for maternal BMI, maternal EPDS scores at gwk 24, and maternal TADS score (Figure 2).

The CC was used to visualize the associations (Figure 3). These ROIs were used for further analysis and confirmed the associations found in the whole-brain statistical models for the genu (β = 0.00106; 95% CI, 0.00046-0.00166; *P* < .001), body (β = 0.00072; 95% CI, 0.00004-0.00140; *P* = .04), and splenium (β = 0.00095;95% CI, 0.00006-0.00185; *P* = .04) (model 1) (Table 2). The regression analysis with the sex interaction included showed that the associations between TADS and regional FA values were slightly steeper for girls, but the interactions were not statistically significant (model 2) (Table 2). Last, after controlling for child sex, child age from conception, child age from birth, maternal prenatal stress measured using the EPDS at gwk 24, maternal TADS score, maternal education, maternal BMI, maternal age, and paternal education, the associations between paternal TADS sum scores and infant CC regional FA values in the genu (β = 0.00096; 95% CI, 0.00034-0.00158;, *P* = .003) and splenium (β = 0.00090; 95% CI, 0.00000-0.00180; *P* = .049) remained statistically significant (model 3) (Table 2). In the body of the CC, the *P* value did not reach statistical significance (β = 0.00061; 95% CI, −0.00008 to 0.00127, *P* = .08).

**Table 2.** Regression Analyses With Corpus Callosum Fractional Anisotrophy Values as the Dependent Variables

Corpus callosum subregion	β (95% CI)	Δ*r*^2^	*P* value
**Model 1: TADS**			
Genu	0.00106 (0.00046 to 0.00166)	0.150	<.001
Body	0.00072 (0.00004 to 0.00140)	0.059	.04
Splenium	0.00095 (0.00006 to 0.00185)	0.060	.04
**Model 2: TADS child sex interaction**			
Genu	0.00034 (−0.00089 to 0.00157)	0.004	.58
Body	0.00123 (−0.00013 to 0.00258)	0.046	.08
Splenium	0.00128 (−0.00053 to 0.00309)	0.028	.16
**Model 3: Fully adjusted TADS**^a^			
Genu	0.00096 (0.00034 to 0.00158)	0.139	.003
Body	0.00061 (−0.00008 to 0.00127)	0.050	.08
Splenium	0.00090 (0.00000 to 0.00180)	0.064	.049

Abbreviations: BMI, body mass index; EPDS, Edinburgh Postnatal Depressive Scale; TADS, Trauma and Distress Scale.

^a^ Covariates: child sex, child age from conception, child age from birth, maternal prenatal stress measured using the EPDS, maternal TADS sum score, maternal education, maternal BMI, maternal age, and paternal education.

## Discussion

The results of this cohort study suggest that paternal ELS is associated with offspring white-matter development in early infancy. These associations remained significant after controlling for maternal ELS and many key pregnancy-related factors. To the best of our knowledge, this is the first report of these findings for humans.

The CC and especially the genu of the CC was one of the brain areas where the association was the strongest. This is an area in the brain that matures early. Importantly, prior research has found this region to be affected by ELS.^30,31,32^ From a methodological validity perspective, the CC can be distinguished reliably within all DTI images,^33^ whereas coregistrations used in the TBSS preprocessing can present a challenge with the less developed white-matter tracts toward peripheral brain areas.^34^ Additionally, the CC is one of the few regions of the brain where a tensor model is an appropriate fit to the data.^35^ These details lend confidence to the robustness of our findings.

It is often assumed that higher FA values correspond to a more mature brain, as an increase in FA values has been observed throughout childhood and adolescence.^36^ Some evidence suggests that a more advanced developmental stage or maturation may predispose to autism.^37^ However, accelerated brain development could be an intergenerational adaptation to the paternal adverse environment and thus could increase the probability of offspring survival.

The findings may be explained by direct paternal genetic transmission of brain development or paternal gene-environment correlation. However, these explanations present difficulties in incorporating the fact that traumatic events were associated with the brain FA values in a cumulative way, although at least 1 study^38^ has suggested that some genetic components might predispose children to experience childhood abuse and neglect, suggesting a gene environment correlation. Another possibility is that the association is mediated through male germ line epigenetic modifications, a mechanism that has been demonstrated in animal experiments.^9,10^ This suggestion is supported by the fact that the association was robust and survived controlling for many potential confounders.

### Limitations

This study has some limitations. A causal relationship between paternal ELS and newborn brain function cannot be assumed without epigenetic data. Thus, these findings should be considered as suggestive of a relationship that should be replicated in other studies. Also, the relatively small sample size should be mentioned as a limitation.

## Conclusion

Although only a follow-up of the children will allow us to eventually address the question of the functional consequences of our findings, the results of this cohort study still point to the possibility that this brain phenotype in the child may be inherited from the father and is associated with his ELS experience. As the effects of early stress have been implied in the mechanisms of several health conditions, these findings may have wide-reaching implications.
